# Affinity Peptide-Based Circularly Permuted Fluorescent Protein Biosensors Loaded in a Microfluidic System for Systemic Lupus Erythematosus Diagnosis

**DOI:** 10.3390/s26103024

**Published:** 2026-05-11

**Authors:** Shuai Shao, Zerui Yang, Jiaqi Liu, Zhi Li, Bo Liu

**Affiliations:** 1 Central Hospital, Dalian University of Technology, Dalian 116021, China; shaos@dlut.edu.cn (S.S.); 18846754758@163.com (Z.Y.); ljiaqi@mail.dlut.edu.cn (J.L.); 2 Liaoning Key Lab of Integrated Circuit and Biomedical Electronic System, Faculty of Medicine, Dalian University of Technology, Dalian 116024, China

**Keywords:** systemic lupus erythematosus (SLE), anti-dsDNA antibody, circularly permuted fluorescent protein (cpFP), phage display, microfluidic chip

## Abstract

Systemic lupus erythematosus (SLE) is a chronic autoimmune disease, with anti-double-stranded DNA (anti-dsDNA) antibodies as its serological biomarkers. However, conventional anti-dsDNA antibody detection methods, which mainly rely on antibody-binding assays, often suffer from limited sensitivity and specificity, cumbersome procedures, and poor suitability for accurate clinical analysis. Herein, we developed an integrated detection system combining a circularly permuted fluorescent protein (cpFP)-based biosensor with a microfluidic chip for rapid and reliable anti-dsDNA antibody detection. The biosensor, cpR-dsAb-C1, was engineered from mApple by inserting an affinity peptide identified through phage display, enabling specific recognition of the variable region of anti-dsDNA antibodies. The biosensor exhibited good sensitivity, specificity, and anti-interference capability. Furthermore, integration of cpR-dsAb-C1 with a polydimethylsiloxane (PDMS)-based microfluidic chip yielded a microfluidic detection platform with good linearity for rapid antibody analysis. Clinical validation showed significantly higher anti-dsDNA antibody levels in patients with SLE than in healthy controls, and the results were consistent with those obtained using routine clinical methods, with an accuracy exceeding 95%. Overall, this system provides a promising low-cost, efficient, and accurate strategy for the early diagnosis and dynamic monitoring of SLE.

## 1. Introduction

Systemic lupus erythematosus (SLE) is a chronic multisystem autoimmune disease with a complex etiology [1]. The core pathology of SLE involves aberrant activation of the immune system, leading to autoantigen recognition, widespread inflammation, and multi-organ damage [2]. The disease is characterized by highly heterogeneous clinical manifestations, an insidious onset, and a high recurrence rate, posing a serious threat to patients’ health and survival. Early diagnosis and dynamic disease monitoring are crucial for improving patient outcomes, delaying disease progression, and reducing the healthcare burden. Biomarker detection remains a major diagnostic approach for SLE, among which autoantibody markers are the most widely used indicators [3]. Anti-double-stranded DNA (anti-dsDNA) antibody is the highest-weighted specific biomarker in the immunological domain of the 2019 EULAR/ACR classification criteria for SLE because of its high specificity (up to 96%) [4]. Previous studies have demonstrated that serum anti-dsDNA antibody levels are significantly correlated with SLE development [5]. Notably, anti-dsDNA antibody can be detected during the subclinical stage, making it a crucial biomarker for the early diagnosis of SLE [6]. Anti-dsDNA antibody has irreplaceable value throughout the entire process of disease diagnosis and treatment. However, traditional detection methods used in clinical practice, such as ELISA and indirect immunofluorescence, are mainly dependent on antigen–antibody reactions, which results in defects including low sensitivity, insufficient specificity, cumbersome and time-consuming procedures, the requirement for batch processing, and poor stability [7]. Therefore, it is crucial to develop novel and highly efficient anti-dsDNA antibody detection technologies that surpass current methods based on antibody binding reactions for clinical diagnosis and treatment.

Genetically encoded fluorescent biosensors have shown great potential for the visualization and measurement of biochemical parameters [8], particularly those based on circularly permuted fluorescent protein (cpFP). CpFP is a novel fluorescent sensing element engineered through directed protein modification. Rearrangement of the amino acid sequence of conventional fluorescent proteins restructures the fluorophore, allowing the fluorescent signal to be activated and amplified only when the binding arm within the cpFP biosensor binds to its target and adopts a stable and complete conformation [9]. CpFP biosensors offer distinct advantages, including high sensitivity, strong specificity, ease of use, and excellent biocompatibility, demonstrating broad application potential in the field of biosensing [10,11]. Considering that cpFP biosensors detect targets without depending on antigen–antibody reactions, cpFP biosensors are likely to overcome the limitations of current detection methods in clinical practice.

In this study, we addressed the defects of current clinical detection methods of anti-dsDNA antibody and proposed a novel strategy with a cpFP biosensor as the core technology to overcome the limitations of antigen–antibody reactions and improve the detection of anti-dsDNA antibody. The results indicated that the cpFP biosensor, cpR-dsAb-C1, detected anti-dsDNA antibody with high sensitivity and outstanding specificity. Loading cpR-dsAb-C1 into a microfluidic chip established a detection platform that enabled miniaturized serum analysis and performed accurately in clinical sample testing. The study expects that the integrated detection platform will be reliable, low-cost, and easy to operate for quantitative measurement of anti-dsDNA antibody concentrations, as well as non-laboratory rapid detection, to advance SLE diagnosis and monitoring.

## 2. Materials and Methods

### 2.1. Phage Display

The variable region of the heavy chain of an anti-dsDNA antibody (amino acid sequence: SRFIFSSYAMSWVRQAPGKGLEWVSGISGTGGSTYYADSVKGR) [12] was synthesized and used as the target molecule for phage display screening. A random 12-peptide phage display library (E8110SC, New England BioLabs, Ipswich, MA, USA) was used to identify peptides with specific affinity for the target through solid-phase biopanning. Biopanning was performed for three consecutive rounds with progressively increased selection stringency. In each round, the synthesized target molecule was immobilized on a solid phase, followed by a blocking step to reduce non-specific binding. The phage display library was then added and incubated with the immobilized target to allow specific binding of phage-displayed peptides. After incubation, unbound and non-specifically bound phages were removed by means of washing, and the specifically bound phages were subsequently eluted and amplified for use in the next round of selection. In the first round of screening, washing was carried out using TBST buffer. To further improve the specificity of enrichment, the Tween-20 concentration in the TBST washing buffer was increased to 0.5% in the second and third rounds, thereby increasing the washing stringency. The recovered phage titers from each round were determined via plaque assay to monitor enrichment efficiency during the screening process. After the third round of biopanning, 30 individual blue plaques were randomly selected from the output plates and amplified separately in bacterial culture. The resulting phage stocks were purified and subjected to DNA sequencing. Based on the sequencing results, four candidate affinity peptide sequences were identified and selected for subsequent validation experiments.

### 2.2. Peptide Synthesis

The heavy-chain variable region of the anti-dsDNA antibody and the affinity peptides identified from the phage display screening were synthesized by Synpeptide Co., Ltd. (Shanghai, China) using the standard solid-phase fluorenylmethoxycarbonyl (Fmoc) method. The synthesized peptides were purified by means of high-performance liquid chromatography (HPLC) to a purity of at least 95% and characterized via mass spectrometry (MS).

### 2.3. Molecular Docking

Homology modeling and molecular docking were performed to predict the binding modes and binding stability between the affinity peptides and the anti-dsDNA antibody. First, a homology search of the screened affinity peptide sequences against the Protein Data Bank (PDB) database was conducted using the NCBI Protein BLAST (version 2.16.0) tool. The top four proteins with the highest homology scores were selected as templates for structural modeling. Subsequently, Modeller 10.2 software was used to construct the three-dimensional structures of the target affinity peptides. The optimal peptide conformation was selected based on the highest GA341 score and the lowest discrete optimized protein energy (DOPE) value. The crystal structure of the anti-dsDNA antibody (PDB ID: 6uta) was obtained from the PDB database and preprocessed using Discovery Studio software, including removal of water molecules and addition of hydrogen atoms, prior to use as the receptor. Molecular docking was then carried out using HEX software with the Shape + Electro + DAR composite algorithm. The optimized affinity peptide structure was used as the ligand, and the anti-dsDNA antibody was used as the receptor. Binding stability was evaluated according to the predicted binding free energy. In addition, PyMOL software (version 2.6.0) was used to visualize the molecular interaction patterns between the peptide ligand and the antibody receptor, mainly including polar contacts and hydrogen bond interactions.

### 2.4. ELISA

The binding activity and specificity of the screened affinity peptides were validated via ELISA. A standard anti-dsDNA antibody solution (100 µg/mL; ab27156, Abcam, Shanghai, China) was coated onto 96-well plates at 150 µL per well. Uncoated wells were used as blank controls. Each group was analyzed in triplicate. The plates were incubated overnight at 4 °C to allow protein immobilization. After coating, the wells were blocked with 300 µL of bovine serum albumin (BSA) blocking solution to reduce non-specific binding, followed by pre-incubation with 200 µL of TBS/Tween buffer. Positively infected phage samples were then added to the wells at serial dilutions ranging from 1 × 10^12^ to 2 × 10^5^ PFU/well. After incubation at room temperature for 1–2 h, the wells were washed six times with TBST buffer to remove unbound phages. Subsequently, 200 µL of horseradish peroxidase (HRP)-conjugated anti-M13 antibody (11973-MM05T-H, YQShengzhou, Beijing, China), diluted 1:5000, was added to each well and incubated at room temperature for 1 h. After additional washing, 200 µL of ABTS chromogenic solution (HY-15902, MedChemExpress LLC, Monmouth Junction, NJ 08852, USA) was added to each well and incubated at room temperature for 10–60 min. Absorbance was measured at 405–415 nm using a microplate reader to evaluate the binding of the affinity peptides to the anti-dsDNA antibody.

### 2.5. BLI

Real-time detection of the binding kinetic parameters between the screened affinity peptides and the anti-dsDNA antibody was performed by means of bio-layer interferometry (BLI) using a Fortebio Octet RH96 instrument (Sartorius AG, Goettingen, Germany). First, the standard anti-dsDNA antibody was biotinylated at a biotin-to-protein molar ratio of 3:1. After purification using a desalting column, the protein concentration and recovery yield were determined by measuring absorbance at 280 nm. Super Streptavidin (SSA) biosensors were pre-wetted in PBS buffer for more than 10 min before analysis. Two biosensors were used for immobilization of the biotinylated antibody, and two additional biosensors were used as parallel reference controls. After antibody immobilization, the assay steps, including baseline acquisition, association, dissociation, and equilibrium analysis, were configured in the instrument software. The antibody-immobilized biosensors were then exposed to affinity peptide samples at a series of concentrations, and changes in the interference signal were recorded continuously in real time. The resulting data were fitted using the supporting software to determine the association rate constant (Ka), dissociation rate constant (Kd), and equilibrium dissociation constant (KD). These parameters were used to evaluate the binding specificity, affinity, and kinetic characteristics of the affinity peptides toward the anti-dsDNA antibody.

### 2.6. Plasmid Construction

mApple, a red circularly permuted fluorescent protein (cpFP), was used as the template for biosensor engineering. The screened affinity peptide was split at its midpoint and fused separately to the N- and C-termini of mApple through flexible GS linkers. The resulting recombinant DNA sequence was cloned into the pRSET-B plasmid using Asc I and Cla I restriction sites.

### 2.7. Recombinant Protein Expression and Purification

Plasmids containing the constructed cpFP biosensor sequence were transformed into *E. coli* BL21 (DE3) cells for recombinant protein expression. Single colonies were inoculated and cultured until the optical density at 600 nm (OD600) reached 0.8, after which isopropyl β-D-1-thiogalactopyranoside (IPTG) was added to a final concentration of 1 mM. Protein expression was induced for 4 h at 16 °C to promote proper folding of the soluble protein. Following induction, the bacterial cells were harvested via centrifugation at 10,000 rpm for 15 min and resuspended in lysis buffer containing 10 mM imidazole, 50 mM NaH_2_PO_4_, and 300 mM NaCl (pH 7.4) at a ratio of 1:10 (*w*/*v*). The cell suspension was then sonicated on ice. After centrifugation at 10,000 rpm and 4 °C for 20–30 min, the supernatant containing the soluble protein fraction was collected. The cpFP biosensor protein was purified using an AKTA protein purification system equipped with a Ni-NTA affinity column. The eluted protein was subsequently dialyzed overnight against PBS at 4 °C to remove residual impurities, including imidazole. The purified protein was stored at −20 °C in the dark for subsequent experiments.

### 2.8. Fluorescence Detection and Analysis

A reaction system consisting of 180 μL of biosensor solution (final concentration, 50 ng/mL) and 20 μL of standard anti-dsDNA antibody solution (final concentration, 600 ng/mL) was used to evaluate the sensitivity, specificity, and stability of the biosensor. Bovine serum albumin (BSA) and goat anti-mouse secondary antibody were used as non-binding protein controls. The same reaction system was used for linearity validation, except that the final concentration of the standard anti-dsDNA antibody was varied as required. Full-wavelength fluorescence scanning was performed using a microplate reader with excitation at 490 nm and emission collected from 550 to 690 nm at 10 nm intervals. All samples were analyzed in triplicate to ensure experimental reproducibility. The experimental data were processed based on the fluorescence intensity measured at 600 nm to quantify changes in the fluorescent signal induced by binding between the cpFP biosensor and the anti-dsDNA antibody. The final fluorescence response was expressed as F/F0, where F0 represented the basal fluorescence intensity of the biosensor in PBS.

Fluorescence images were acquired using an inverted microscope (Nikon Eclipse Ti Series, Ti-Fl Epi-fl/1, Nikon, Tokyo, Japan) equipped with a cooled charge-coupled device (CCD) camera (EVO-512-M-FW-16-AC, Photometrics, Tucson, AZ, USA), a BP565–585 excitation filter, a DM595 dichroic beamsplitter, and a BA600–690 emission filter for red fluorescence imaging. The exposure time was kept constant for each sample. The fluorescence images were analyzed using a custom MATLAB (version R2024b) script to generate a standard calibration curve correlating fluorescence intensity with the concentration of anti-dsDNA antibody.

### 2.9. Fabrication and Surface Functionalization of Microfluidic Chips

Polydimethylsiloxane (PDMS) prepolymer and curing agent were mixed at a ratio of 10:1 (*w*/*w*), degassed under vacuum, and thermally cured at 80 °C for 1 h to form the bulk polymer. The cured PDMS was then cut and punched to fabricate the chip body. Cover glasses were sequentially cleaned with acetone and anhydrous ethanol, air-dried, and subjected to oxygen plasma treatment (1.5 min, 40–50 Pa) together with the PDMS chip to activate the surfaces for subsequent covalent bonding. The microchannels were modified using a two-step silanization protocol with 3-aminopropyltriethoxysilane (APTES) and glutaraldehyde (GA). Specifically, amino groups were first introduced onto the channel surfaces through treatment with 5% APTES in acetone, followed by curing at 120 °C for 1 h. Aldehyde anchoring sites were then generated through incubation with 5% glutaraldehyde for 30 min. After thorough rinsing and drying, 100 μL of cpFP biosensor solution (50 ng/mL) was introduced into the modified channels and incubated at room temperature for 2 h to enable the covalent immobilization of the cpFP biosensor onto the channel surfaces through aldehyde–amino Schiff base linkages.

### 2.10. Clinical Sample Detection and Performance Evaluation

Clinical samples were collected at the Central Hospital of Dalian University of Technology from January 2025 to April 2025, including 20 patients diagnosed with SLE and 20 healthy volunteers. Serum samples were collected after informed consent had been obtained. Before analysis, each serum sample was diluted 1:10 and then introduced into the microfluidic detection platform. A sample background control was prepared using a mixture containing 20 µL of serum and 180 µL of PBS.

### 2.11. Statistical Analysis

All experimental data were presented as the mean ± standard deviation (SD). Statistical analyses were performed using GraphPad Prism software (version 9.0, GraphPad Software, San Diego, CA, USA). Differences between the two groups were assessed using Student’s *t*-test, whereas comparisons among multiple groups were performed using one-way analysis of variance (ANOVA) followed by the Bonferroni post hoc test. A two-tailed *p*-value < 0.05 was considered statistically significant.

## 3. Results

### 3.1. Phage Display Screening and High-Throughput Identification of Anti-dsDNA Antibody Affinity Peptides

A three-round phage display biopanning procedure was performed using a random dodecapeptide library against the heavy-chain variable region of the anti-dsDNA antibody to identify affinity peptides capable of specifically recognizing and binding to the target antibody. The eluted phage products from each round of selection were serially diluted and plated on LB/IPTG/X-gal agar plates, and fewer than 100 monoclonal blue plaques were counted in each round. Based on the dilution factors, the phage titers were calculated and are summarized in Table A1. As the number of selection rounds increased, both the titer of eluted phages and the phage recovery rate increased progressively, with the eluted phage titer rising from 3.8 × 10^4^ to 6.2 × 10^5^ pfu and the recovery rate increasing from 2.53 × 10^−7^ to 6.2 × 10^−3^. These results indicated effective enrichment of phage clones displaying peptides with high affinity for the anti-dsDNA antibody. Subsequent phage DNA sequencing identified six candidate peptide sequences with specific binding to the anti-dsDNA antibody (Table 1). The high frequency suggested that the peptide was highly retained in the clones after the three-round biopanning and showed higher specificity and affinity for the heavy chain of an anti-dsDNA antibody. Thus, C1–C4 with frequency higher than 1 were selected for subsequent in vitro binding activity validation.

### 3.2. Validation of Affinity Peptide Binding to Anti-dsDNA Antibody and Identification of Optimal Candidates

**Figure 1:**
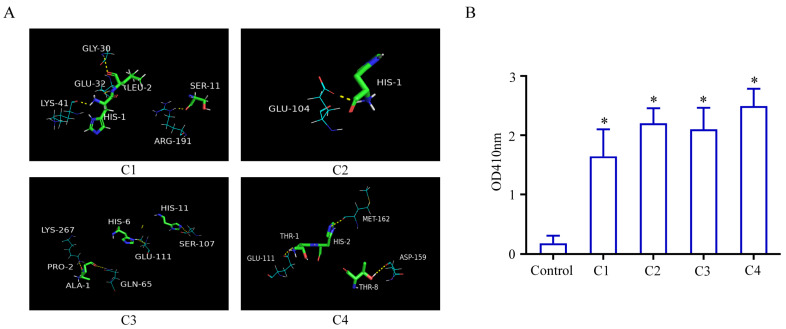
Molecular docking analysis and ELISA validation of affinity peptides binding to anti-dsDNA antibody. (**A**) Molecular docking simulation showing key binding sites and amino acid interactions between affinity peptides (C1, C2, C3, C4) and the anti-dsDNA antibody. The hydrogen bonds formed between the peptides and the antibody are highlighted. (**B**) Binding activity of peptides C1–C4 toward the anti-dsDNA antibody measured by means of ELISA. Results are presented as optical density (OD410) values. Data are expressed as the mean ± standard deviation from four independent biological replicates (n = 4). * *p* < 0.05 compared with the control group.

The four screened high-affinity peptides (C1–C4) were further evaluated via molecular docking, ELISA, and bio-layer interferometry (BLI) assays. Molecular docking analysis, including homology modeling, targeted docking optimization, and binding free energy calculation, was performed to investigate the interaction between peptides and the anti-dsDNA antibody. The results showed that C1 formed four hydrogen bonds with the antibody at four distinct binding sites, with a calculated binding energy of −404.02 kcal/mol (Figure 1A, Table A2). C2 interacted with the antibody through a hydrogen bond at a different binding site compared with C1, with a binding energy of −394.20 kcal/mol. Although C3 and C4 formed a greater number of hydrogen bonds with the antibody, their binding energies were lower than those of C1 and C2. Binding energy is closely related to the hydrogen bond connection, as well as conformational compatibility, hydrophobic interaction, and van der Waals force. Comprehensively assessing the binding energy and hydrogen bonds, C1 and C2 were considered more potent due to the lower binding energies and the effective formation of hydrogen bonds with the anti-dsDNA antibody. In addition, the molecular docking indicated that C1 and C2 bound to the anti-dsDNA antibody in different spatial conformations.

**Figure 2:**
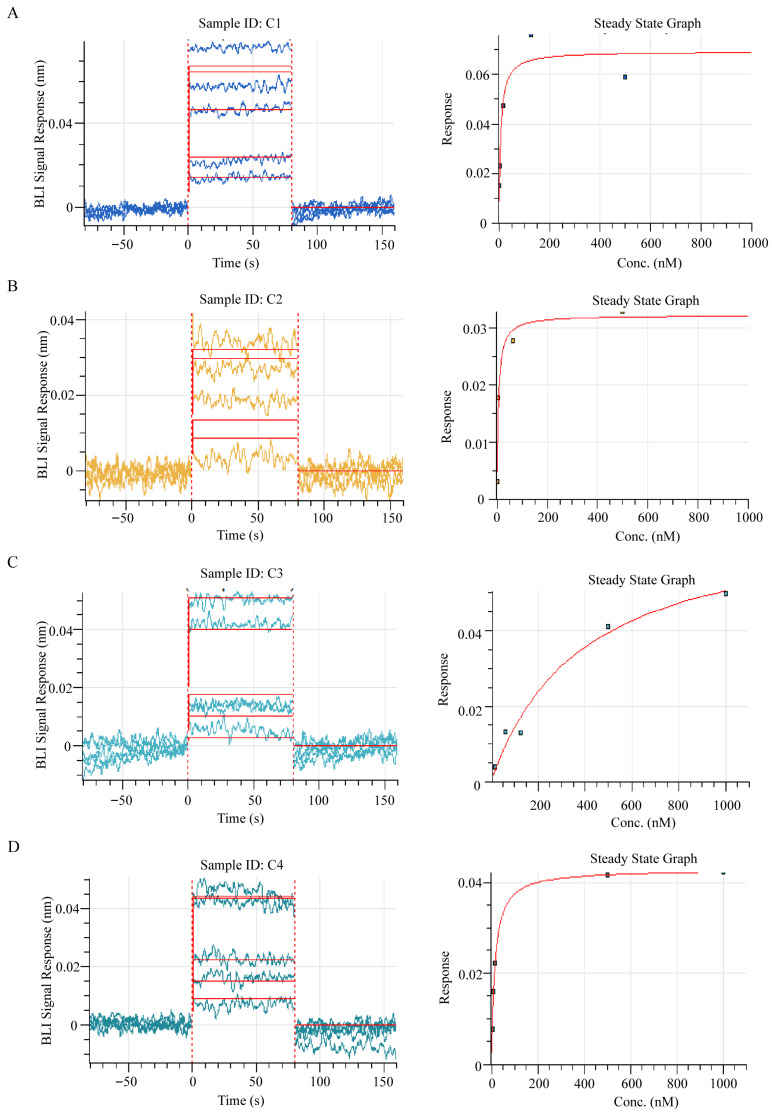
Bio-layer interferometry (BLI) molecular interaction kinetics and affinity analysis of affinity peptides with anti-dsDNA antibody. (**A**–**D**) BLI sensorgrams showing real-time association and dissociation curves for peptides C1, C2, C3, and C4 binding to the anti-dsDNA antibody. The left panels display the BLI signal response (nm) over time (s), with the red dashed boxes marking the association phase. The right panels show the corresponding steady-state binding curves, with the y-axis indicating the steady-state response and the x-axis representing the peptide concentration (nM). The red curves represent the fitted binding models. The equilibrium dissociation constant (KD) was determined via nonlinear fitting of the steady-state data.

To further validate binding activity, phage clones expressing peptides C1–C4 were subjected to ELISA analysis. As shown in Figure 1B, all selected peptides exhibited significant binding to the anti-dsDNA antibody compared with the control group, confirming the effectiveness of the biopanning process. The binding kinetics and affinity were further characterized via BLI. The anti-dsDNA antibody (0.1 µg/mL) was biotinylated, purified via gel filtration desalting, and adjusted to a final concentration of 0.065 µg/mL prior to analysis. The biotinylated antibody was then incubated with gradient-diluted affinity peptides. As shown in Figure 2, all four peptides exhibited specific binding to the anti-dsDNA antibody. Notably, C1 (KD = 7.1× 10^−9^, R^2^ = 0.9223) and C2 (KD = 5.7× 10^−9^, R^2^ = 0.8986) showed significantly higher binding affinities than C3 (KD = 3.9× 10^−7^, R^2^ = 0.9783) and C4 (KD = 1.5× 10^−8^, R^2^ = 0.9975). Taken together, based on the combined results of molecular docking, ELISA, and BLI assays, peptides C1 and C2 were identified as the optimal affinity candidates and selected for subsequent construction of cpFP-based biosensors.

### 3.3. Performance Validation of cpFP Biosensors

**Figure 3:**
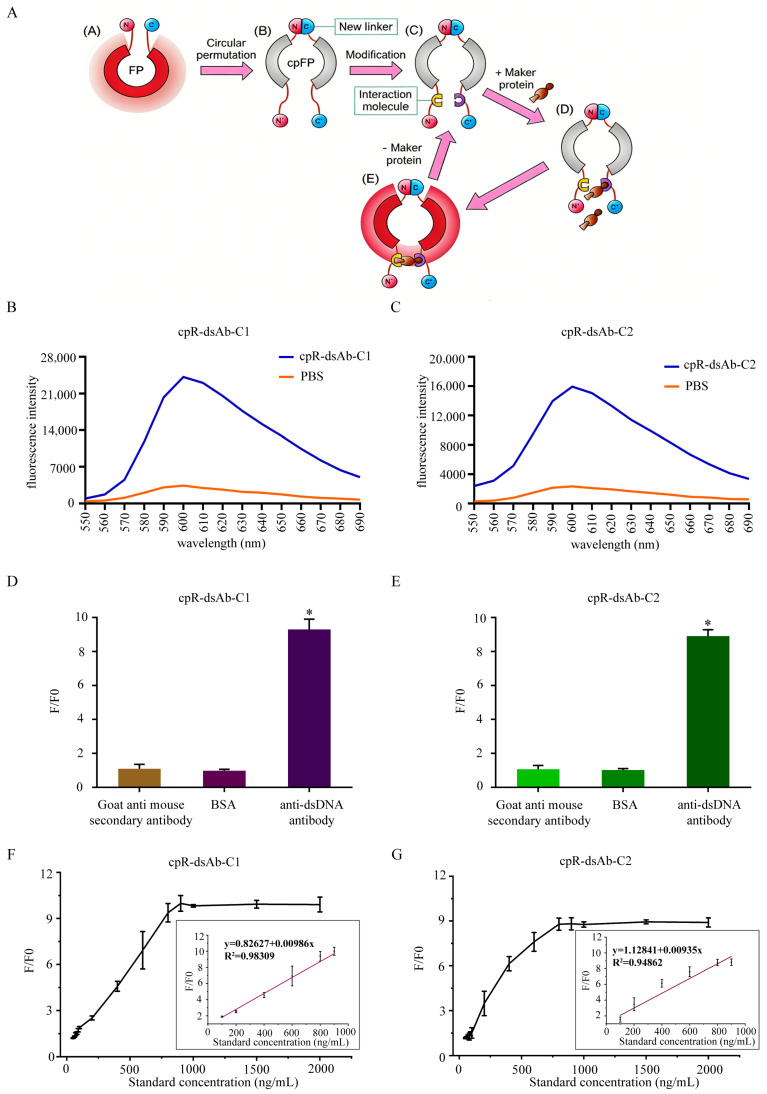
Construction and performance characterization of cpR-dsAb-C1 and cpR-dsAb-C2 biosensors for anti-dsDNA antibody detection. (**A**) Schematic illustration of the design and sensing mechanism of the cpFP-based biosensors. Peptides C1 or C2 were split into two segments and symmetrically inserted into the N- and C-termini of mApple through the GS flexible linker. Binding of anti-dsDNA antibody induced conformational rearrangement of the biosensor and enhanced fluorescence output. (**B**,**C**) Fluorescence emission spectra of cpR-dsAb-C1 (**B**) and cpR-dsAb-C2 (**C**) in the presence of anti-dsDNA antibody (blue) or PBS control (orange). (**D**,**E**) Specificity analysis of cpR-dsAb-C1 (**D**) and cpR-dsAb-C2 (**E**) against anti-dsDNA antibody, goat anti-mouse secondary antibody, and BSA. Data are presented as normalized fluorescence response (F/F_0_) and shown as mean ± SD (n = 5). * *p* < 0.05 compared with the non-target protein groups or control group. (**F**,**G**) Calibration curves of cpR-dsAb-C1 (**F**) and cpR-dsAb-C2 (**G**) for anti-dsDNA antibody detection over the indicated concentration range. The y-axis represents the normalized fluorescence response (F/F_0_) (n = 5). Insets show the corresponding linear regression equations and R^2^ values.

To construct cpFP-based biosensors for anti-dsDNA antibody detection, peptide C1 was split into two segments and symmetrically inserted into the N- and C-termini of mApple through flexible GS linkers, generating the biosensor cpR-dsAb-C1. Using the same strategy, peptide C2 was used to construct cpR-dsAb-C2 (Figure 3A). Fluorescence emission spectra showed that the binding of anti-dsDNA antibody markedly enhanced the fluorescence signals of both biosensors. Specifically, cpR-dsAb-C1 exhibited a 7.8-fold increase in fluorescence intensity at 600 nm, while cpR-dsAb-C2 showed a 7.2-fold increase relative to the PBS control (Figure 3B,C). These results indicate that both biosensors responded to anti-dsDNA antibody with a broad dynamic fluorescence change. These results suggested that the structures of cpR-dsAb-C1 and cpR-dsAb-C2 were able to effectively promote the arrangement of mApple for fluorescence emission by binding to anti-dsDNA antibody.

The specificity of the two biosensors was further evaluated using several substrates, including goat anti-mouse secondary antibody, BSA, and anti-dsDNA antibody (1 µg/mL). As shown in Figure 3D,E, the normalized fluorescence responses (F/F_0_) of both cpR-dsAb-C1 and cpR-dsAb-C2 to anti-dsDNA antibody were significantly greater than those to the non-target proteins, demonstrating good specificity for anti-dsDNA antibody recognition. To assess the quantitative detection performance, anti-dsDNA antibody was tested over a concentration range of 50–2000 ng/mL. For both biosensors, the normalized fluorescence response (F/F_0_) increased progressively with increasing anti-dsDNA antibody concentration, particularly below 1000 ng/mL. Linear regression analysis within this range showed that both CpR-dsAb-C1 and CpR-dsAb-C2 exhibited good linearity, with R2 values greater than 0.9 (Figure 3F,G).

**Figure 4:**
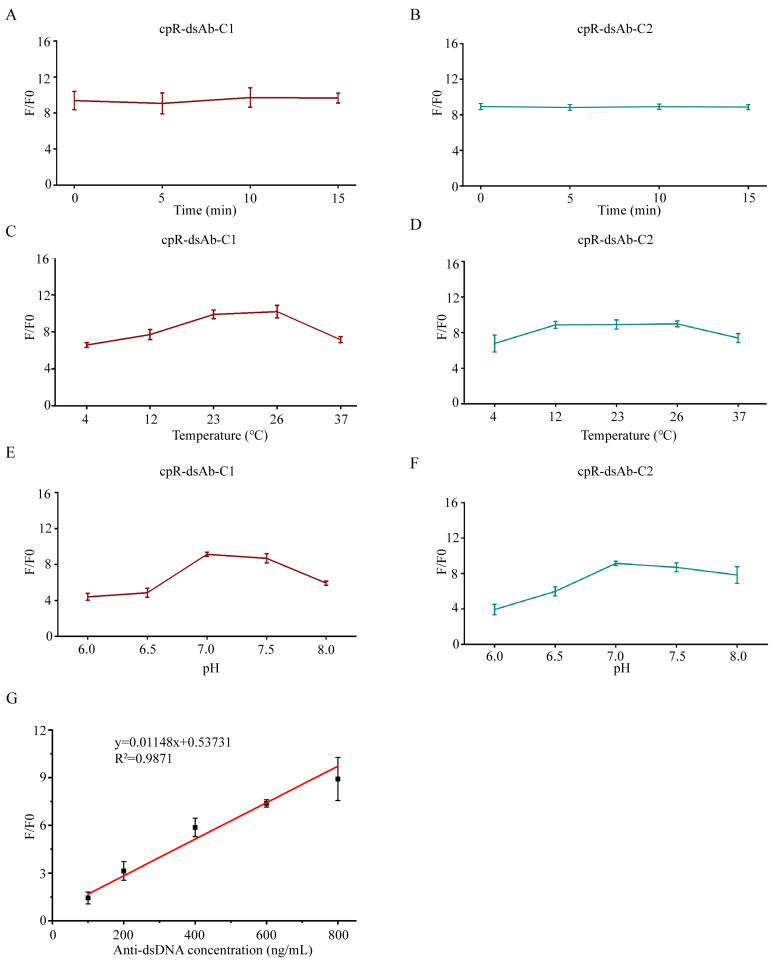
Stability, environmental response, and serum detection performance of cpR-dsAb biosensors. (**A**,**B**) Time-dependent normalized fluorescence responses (F/F_0_) of cpR-dsAb-C1 (**A**) and cpR-dsAb-C2 (**B**) after incubation with anti-dsDNA antibody for 0–15 min. (**C**,**D**) Effects of temperature on the normalized fluorescence responses (F/F_0_) of cpR-dsAb-C1 (**C**) and cpR-dsAb-C2 (**D**), measured at 4–37 °C. (**E**,**F**) Effects of pH on the normalized fluorescence responses (F/F_0_) of cpR-dsAb-C1 (**E**) and cpR-dsAb-C2 (**F**) over the pH range of 6.0–8.0. Data are shown as mean ± SD (n = 5). (**G**) Calibration curve of cpR-dsAb-C1 for anti-dsDNA antibody detection in 10% diluted human serum. The y-axis represents the normalized fluorescence response (F/F_0_). The inset shows the linear regression equation and R^2^ value.

The stability and environmental adaptability of the biosensors were further investigated. Following the addition of anti-dsDNA antibody, both cpR-dsAb-C1 and cpR-dsAb-C2 maintained stable normalized fluorescence responses (F/F_0_) within 15 min (Figure 4A,B). Temperature variation caused only minor fluctuations in the normalized fluorescence response, and both biosensors exhibited optimal performance at room temperature (Figure 4C,D). In addition, the normalized fluorescence response increased as the pH rose from 6.0 to 7.0–7.5 and then declined at higher pH values (Figure 4E,F), suggesting that a near-neutral environment is favorable for biosensor performance.

Overall, these results demonstrate that both cpR-dsAb-C1 and cpR-dsAb-C2 possess desirable dynamic response range, specificity, linearity, and stability for anti-dsDNA antibody detection. In addition, cpR-dsAb-C1 showed measurable normalized fluorescence responses at relatively low anti-dsDNA antibody concentrations, including 50–100 ng/mL. However, because a formal statistical determination of the limit of detection (LOD) and limit of quantification (LOQ) was not performed in the present study, these low-concentration responses are reported only as qualitative evidence of sensitivity rather than as analytically defined detection limits. Therefore, cpR-dsAb-C1 was selected for subsequent serum detection and microfluidic platform integration.

### 3.4. Detection of Anti-dsDNA Antibody in Serum Samples

To further evaluate the practical applicability of cpR-dsAb-C1 in complex biological matrices, healthy human serum was spiked with anti-dsDNA antibody to generate simulated clinical samples with gradient concentrations. A total of 20 µL of serum sample was mixed with 180 µL of cpR-dsAb-C1 at a final concentration of 50 ng/mL after 10-fold dilution of the serum samples. As shown in Figure 4G, the normalized fluorescence response (F/F_0_) of cpR-dsAb-C1 exhibited a favorable linear correlation with anti-dsDNA antibody concentration in 10% human serum. These results indicate that cpR-dsAb-C1 has good resistance to serum interference and supports sensitive and quantitative detection of anti-dsDNA antibody in serum samples, highlighting its potential for clinical diagnostic applications.

### 3.5. Construction of a cpR-dsAb-C1-Loaded Microfluidic Detection Platform

**Figure 5:**
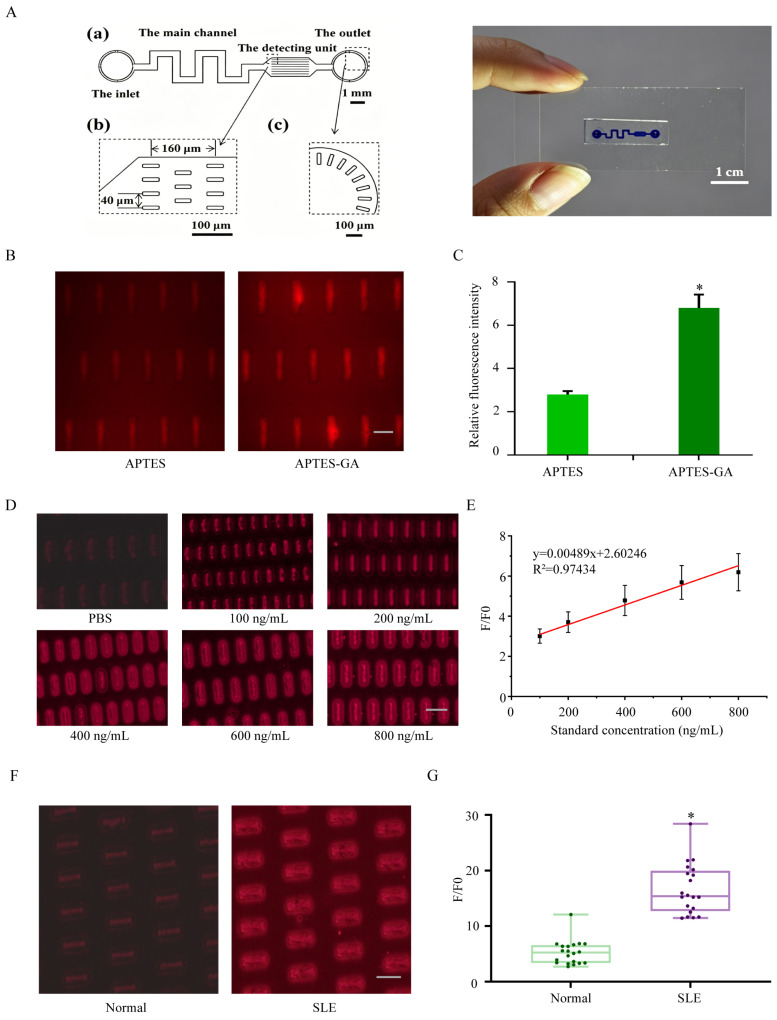
Construction, analytical performance, and clinical serum detection of the APTES-GA- modified microfluidic chip loaded with cpR-dsAb-C1. (**A**) Schematic illustration and photograph of the microfluidic chip, including the overall layout, sensing region, channel geometry, and cross-sectional structure of the functionalized channel. The chip contains a serpentine microchannel and a reaction region for fluorescence detection. Scale bar in the chip photograph [13]. (**B**) Fluorescence images of chip channels after direct APTES modification or APTES-GA coupling modification, with red fluorescence indicating immobilized cpR-dsAb-C1. Scale bar, 100 μm. (**C**) Quantitative analysis of the fluorescence signals in panel B under the two surface modification strategies. Data are shown as mean ± SD (n = 5). * *p* < 0.05. (**D**) Fluorescence images of the optimized microfluidic chip after perfusion with anti-dsDNA antibody standards at different concentrations (PBS, 100, 200, 400, 600, and 800 ng/mL). Scale bar, 100 μm. (**E**) Calibration curve for anti-dsDNA antibody detection using the cpR-dsAb-C1-loaded microfluidic platform, showing a linear relationship between the normalized fluorescence response (F/F_0_) and anti-dsDNA antibody concentration in the range of 100–800 ng/mL. The linear fitting equation is y = 0.00489x + 2.60246, R^2^ = 0.97434. (**F**) Representative fluorescence images of serum samples from healthy volunteers and patients with SLE using the established microfluidic platform. Scale bar, 200 μm. (**G**) Quantitative comparison of normalized fluorescence responses (F/F_0_) between the healthy control group (n = 20) and the SLE group (n = 20). The SLE group exhibited significantly higher fluorescence responses than the healthy control group (* *p* < 0.05).

A microfluidic detection platform was constructed based on the molecule-capture microarray reported in our previous work [13] (Figure 5A). The chip consisted of a serpentine microchannel and a sensing region designed for biosensor immobilization and fluorescence readout. To immobilize cpR-dsAb-C1 on the inner surface of the microchannel, two silanization strategies were evaluated: direct APTES modification and APTES–glutaraldehyde (GA) coupling modification. As shown in Figure 5B,C, the APTES–GA coupling strategy generated significantly stronger fluorescence signals than direct APTES modification (*p* < 0.05, n = 5), indicating more efficient immobilization of cpR-dsAb-C1 on the channel surface. This result is attributable to the conversion of the aminated surface into a highly reactive aldehyde-functionalized surface by glutaraldehyde, which facilitates covalent attachment of the biosensor through Schiff base formation. Therefore, APTES-GA coupling modification was selected for the subsequent fabrication of the microfluidic detection platform.

The analytical performance of the APTES–GA-modified microfluidic platform was then evaluated using anti-dsDNA antibody standards. Serum samples (20 µL) spiked with anti-dsDNA antibody at different concentrations (100, 200, 400, 600, and 800 ng/mL) were diluted 10-fold and infused into the microchannels. PBS was used as the control for normalization of fluorescence signals. As shown in Figure 5D, cpR-dsAb-C1 immobilized in the detection platform effectively reacted with the anti-dsDNA antibody in the mixture to yield strong fluorescence. At 800 ng/mL, the normalized fluorescence response (F/F_0_) was approximately 6.19-fold higher than that of the PBS control. Linear fitting analysis demonstrated a favorable linear relationship between the normalized fluorescence response (F/F_0_) and anti-dsDNA antibody concentration over the range of 100–800 ng/mL (Figure 5E). The corresponding calibration equation is shown in Figure 5E. Collectively, these results confirm that the APTES-GA-modified microfluidic platform enables effective immobilization of cpR-dsAb-C1 and supports quantitative detection of anti-dsDNA antibody.

### 3.6. Detection of Anti-dsDNA Antibody in Clinical Serum Samples

The performance of the microfluidic detection platform was further validated using clinical serum samples from healthy volunteers and patients with SLE. Serum samples from the healthy control group (n = 20) and the SLE group (n = 20) were diluted 10-fold, infused into the detection platform, incubated for 10 min, and then subjected to fluorescence imaging. The fluorescence signals obtained from the images were normalized to the PBS control to generate the normalized fluorescence response (F/F_0_). The corresponding anti-dsDNA antibody concentrations were estimated using the calibration function established in Section 3.5. Representative fluorescence images are shown in Figure 5F, and the quantitative results are summarized in Figure 5G. The healthy control group (n = 20) exhibited a mean normalized fluorescence response of 5.42 ± 2.24, whereas the SLE group (n = 20) showed a significantly higher value of 16.64 ± 4.29 (* *p* < 0.05). Based on the calibration equation, the estimated anti-dsDNA antibody concentration was 576.18 ± 74.12 ng/mL in healthy volunteers and 2870.66 ± 345.10 ng/mL in patients with SLE. These results demonstrate that the cpR-dsAb-C1-loaded microfluidic platform can distinguish SLE serum samples from healthy controls and has potential for anti-dsDNA antibody analysis in clinical specimens.

## 4. Discussion

CpFP biosensors have been widely used for live-cell imaging and the detection of biomolecules and ions, such as Ca^2+^ [14], Hg^2+^ [15], α-melanocyte-stimulating hormones [16], and glutarate [17]. Although these cpFP biosensors have demonstrated the strong capabilities of cpFP-based platforms for detecting small molecules, the detection of macromolecular proteins remains challenging because of the underlying sensing mechanism. Detection by cpFP biosensors relies on the switching of fluorescence signals between the “on” and “off” states, which is induced by the “recovery” and “destruction” of the fluorophore structure. Inhibiting fluorescent signals by destroying the fluorophore structure for detection is an acceptable principle for the cpFP biosensor, but increasing fluorescence via recovery of the fluorophore structure is more common. According to this principle, the native N- and C-termini of a cpFP are reconnected, and new termini are generated, inhibiting the fluorescence emission. A binding arm is linked to the new termini by a linker. When the binding arms recognize and detect the target molecule, the cpFP structure rearranges to recover the fluorescence. However, the spatial conformation of cpFP restricts the length of the binding arm. Therefore, designing length-constrained binding arms that can specifically and tightly capture macromolecular proteins remains an urgent challenge.

Peptides are typically short linear chains composed of fewer than 50 amino acid residues. Because they lack complex tertiary structures, their functions are primarily determined by their amino acid sequences [18]. Affinity peptides screened via phage display exhibit high affinity and specificity, making them suitable as binding arms in cpFP biosensors. Our lab has developed a series of cpFP biosensors for lung cancer biomarkers using affinity peptides identified through phage display [19,20]. Considering that mutations in the N-terminal portion of the antibody variable region contribute to antibody diversity [21], the variable region of the anti-dsDNA antibody was used as the target for phage display screening to improve specificity. Based on monoclonal sequencing, molecular docking, ELISA and BLI validation, C1 and C2 were ultimately identified as high-affinity, highly specific peptides. These peptides showed high occurrence frequency, low dissociation constants, and excellent binding capacity. These features enable C1 and C2 to recognize anti-dsDNA antibody specifically and bind to the antibody tightly. Notably, only a limited short sequence in the variable region of the heavy chain of an anti-dsDNA antibody was detected by an affinity peptide, suggesting the potential binding sites were spatially close. When the affinity peptide was separately cleaved in the middle into two six-peptides and linked to the new N- and C- termini, the six-peptides bind to different but close sites of anti-dsDNA antibody, respectively. The combination ensured that the new C-terminal and N-terminal were also spatially close to each other, which recovered the cpFP conformation to yield strong fluorescent signals. According to this strategy, the two constructed biosensors, cpR-dsAb-C1 and cpR-dsAb-C2, functioned as expected, with cpR-dsAb-C1 showing particularly superior performance. These results demonstrate that screening affinity peptides via phage display against specific regions of macromolecular proteins is a reliable strategy for developing binding arms in cpFP biosensors.

As demonstrated by the evaluation results, cpR-dsAb-C1 exhibited a dynamic response range of more than seven-fold toward anti-dsDNA antibodies within 15 min. CpR-dsAb-C1 showed no obvious cross-reactivity with non-target proteins, with a low detection limit of 50 ng/mL, defined as the lowest tested concentration that produced a measurable normalized fluorescence signal, and a linear detection range of 50–900 ng/mL. The biosensor also retained excellent linearity in 10% diluted serum matrices, further demonstrating its outstanding specificity. As a protein-based biosensor, the performance of cpR-dsAb-C1 inevitably showed slight fluctuations with changes in pH and temperature. The reporting of cpR-dsAb-C1 to anti-dsDNA antibody depended on its conformational recovery by binding to the anti-dsDNA antibody. However, the protein conformation was impacted by pH and temperature. Unsuitable pH and temperature may change the conformation of the cpFP biosensor by affecting the formation of hydrogen bonds. PH also changes the charge distribution on the cpFP biosensor and further destroys the electrostatic attraction between the cpFP biosensor and targets, which causes a decline in the performance of the cpFP biosensor. Nevertheless, its optimal performance was achieved at 26 °C and pH 7.0, which is acceptable considering that serum samples are nearly neutral and most detection scenarios are conducted at room temperature.

Several studies currently report different detection methods for anti-dsDNA antibody. For instance, Li et al. developed a magnetic bead-based immunofluorescence assay (IFA) for anti-dsDNA detection [22]. Synthetic dsDNA was coated on magnetic beads using a biotin/streptavidin assay. Anti-dsDNA antibody in the sample was enriched on the beads. FAM-labeled secondary antibody was used to report the detection by yielding a strong fluorescent signal. Pablo et al. proposed an electrochemical biosensor with synthetic dsDNA immobilized on the surface of a disposable screen-printed carbon graphite electrode [23]. Secondary IgG antibodies conjugated to the electroactive enzyme HRP form a sandwich with dsDNA and anti-dsDNA antibody. The electrochemical reduction of the oxidized TMB generated through the catalysis of HRP is measured, which is dependent on the amount of anti-dsDNA antibodies located on the surface of the sensor. Lei et al. engineered a rapid vertical flow assay (VFA) to detect anti-dsDNA antibody [24]. Anti-dsDNA binds to the dsDNA on a nitrocellulose membrane (NCM) and forms an antigen–antibody immunocomplex. Biotinylated anti-human IgG is then added to bind with the anti-dsDNA. Finally, streptavidin-conjugated gold nanoparticles (GNPs) are added to bind with the anti-IgG antibody, displaying a green colorimetric signal on the NCM. Although these studies employed different carriers for detection, the core principle remained the antigen–antibody reaction. Thus, these methods can be regarded as optimizations based on ELISA and limited by the same cues. For instance, double incubation resulted in a complicated operation. The reactions between dsDNA and anti-dsDNA antibody and between anti-dsDNA and secondary antibody increased the testing time. In addition, the synthetic dsDNA and secondary antibody increased the cost of detection. Compared with the methods based on antigen–antibody reaction, cpR-dsAb-C1 allowed label-free real-time monitoring, which saved the cost of secondary antibodies and the time of single incubation. Integrating the cpFP biosensor into a microfluidic system further simplified operations and lowered the detection requirement to specialist equipment.

Briefly, the detection platform enables the detection of anti-dsDNA antibody without depending on the antigen–antibody reaction, lowering the detection cost and time. The fluorescence differences of the microfluidic detection platform reacting to SLE and healthy samples were significant enough for observation by the naked eye. Thus, the microfluidic detection platform could also be suitably applied in primary medical institutions, point-of-care rapid testing scenarios, and non-laboratory detecting settings, in addition to quantitative analysis. The features of simple operations and direct result reading make the detection platform likely to evolve into a point-of-care-testing (PoCT) tool to facilitate the early diagnosis and home monitoring of SLE.

However, the integrated detection platform still possesses some limitations. First, clinical SLE evaluation usually combines with complement C3/C4, inflammatory factors, and related clinical symptoms besides anti-dsDNA antibody. The current detection platform only allows one indicator of the anti-dsDNA antibody test. Although the platform reserved sufficient expansion space for multi-marker combined detection. How to integrate detection methods with other SLE biomarkers into the detection platform is a worthy consideration. A potentially feasible method is constructing cpFP biosensors for other SLE biomarkers and loading biosensors into a microfluidic module. Each module supports the detection of one specific biomarker and is further integrated into a detection platform that is paired with a multichannel, which allows simultaneous detection of multi-markers. Setting a pre-treatment modulation that separates and dilutes the serum from the blood collected by the patient themselves is also necessary. Additionally, the final results detected via cpR-dsAb-C1 were given in nanograms per mL, but the clinical detection results are reported in international units per mL (IU/mL). Since there is no recognized conversion factor between the two units, assessing the agreement between cpR-dsAb-C1 and other assays still needs to be explored. Optimizing the detection process to establish a standard protocol and expanding the sample size to confirm the cut-off for cpR-dsAb-C1 seems to be necessary in future work. The positive or negative results determined by the cut-off enable the performance of the detection platform to be compared in terms of specificity, accuracy, and agreement with other detection methods.

## 5. Conclusions

This study successfully leveraged the combined advantages of affinity peptides and cpFP to engineer a novel biosensor, cpR-dsAb-C1, for the detection of anti-dsDNA antibody. CpR-dsAb-C1 exhibited high sensitivity and specificity toward anti-dsDNA antibody. By integrating cpR-dsAb-C1 with a PDMS microfluidic chip, we established a microfluidic detection platform that enabled rapid and quantitative detection of anti-dsDNA antibody and effectively distinguished SLE patients from healthy volunteers with simple operation. The total assay time of 10 min, the few manipulation steps, and the direct reading of results will aid the application of the platform outside of laboratory facilities and promote the platform to evolve into a point-of-care-testing (PoCT) tool to promote the early diagnosis and home monitoring of SLE.

## Figures and Tables

**Table 1:** Peptide sequences identified through phage display screening against the anti-dsDNA antibody.

Clone	Amino Acid Sequence	Frequency
C1	HLHVGHSHHNSI	11
C2	HTRIPHHSTHWL	6
C3	APRLSHHIAHHH	3
C4	THPHWHHTIYHN	2
C5	TVHACCVPNHHL	1
C6	SYFHHPWHRAMH	1

## Data Availability

The raw data supporting the conclusions of this article, which are not publicly available, will be made available by the authors, without undue reservation.

## Appendix A

**Table A1:** Phage titer and recovery rate during three rounds of biopanning (pfu/10 μL).

Screening Round	Input Quantity	Output Quantity	Recovery Rate (Output/Input)
First round	1.5 × 10^11^	3.8 × 10^4^	2.53 × 10^−7^
Second round	1.0 × 10^11^	5.9 × 10^4^	5.9 × 10^−7^
Third round	1.0 × 10^8^	6.2 × 10^5^	6.2 × 10^−3^

**Table A2:** Docking binding site.

Affinity Peptide	Binding Energy	Binding Site	Hydrogen Bond
C1	−404.02	LYS41-HIS1,GLY30-LEU2,LEU2-GLU32,ARG191-SER11	4
C2	−394.20	HIS1-GLU104	1
C3	−289.29	HIS11-SER107,HIS6-GLU111,ALA1-GLN65,PRO2-LYS267	4
C4	−358.23	THR8-ASP159,HIS2-MET162,THR1-GLU111	3
